# Characteristics of ocular trauma in the United States

**DOI:** 10.5935/0004-2749.20220035

**Published:** 2022

**Authors:** Catherine H. He, David M. Poulsen, Afshin Parsikia, Joyce N. Mbekeani

**Affiliations:** 1 Albert Einstein College of Medicine, Bronx, NY, USA; 2 Casey Eye Institute, Oregon Health and Science University, Portland, OR, USA; 3 Department of Surgery (Trauma), Jacobi Medical Center, Bronx, NY, USA; 4 Research Services, University of Pennsylvania, PA, USA; 5 Department of Surgery (Ophthalmology), Jacobi Medical Center, Bronx, NY, USA; 6 Department of Ophthalmology & Visual Sciences, Albert Einstein College of Medicine, Bronx, NY, USA

**Keywords:** Eye injuries, Blindness/prevention & control, United Sates/epidemiology, Traumatismo ocular, Cegueira/prevenção & controle, Estados Unidos/epidemiologia

## Abstract

**Purpose:**

We aimed to study the characteristics of ocular trauma, an important but largely preventable global cause of blindness, in the United States.

**Methods:**

Retrospective chart review of the National Trauma Data Bank (2008-2014) was performed. All patients with ocular trauma were identified using ICD-9CM codes. The collected data were statistically analyzed with student’s t-test, Chi-squared test, and logistic regression analysis performed using the SPSS software. The significance was set at p<0.05.

**Results:**

It was found that 316,485 (5.93%) of the 5,336,575 admitted trauma patients had ocular injuries. Their mean (SD) age was 41.8 (23) years, and most of them were men (69.4%). Race/ethnicity distribution was White 66.1%, Black 15.1%, and Hispanic 12.3%. The common injuries were orbital 39.5% and eye/adnexa contusions 34%. Associated traumatic brain injury was present in 58.2%. The frequent mechanisms were falls 25.5%, motor vehicle accident-occupant 21.8%, and struck by/against 17.6%. Patients <21 years of age had higher odds of cut/pierce injuries (OR=3.29, 95%CI=3.07-3.51) than the other age groups, those aged 21-64 years had higher odds of motor vehicle accident-cyclist (OR=4.95, 95%CI=4.71-5.19), and those >65 years had higher odds of falls (OR=16.75, 95%CI=16.39-17.12); p<0.001. The Blacks had a greater likelihood of firearm injuries (OR=3.24, 95%CI=3.10-3.39) than the other racial/ethnic groups, the Hispanics experienced more of cut/pierce injuries (OR=2.01, 95%CI=1.85-2.18), and the Whites experienced more of falls (OR=2.3, 95%CI=2.3-2.4); p<0.001. The Blacks (OR=3.41, 95%CI=3.34-3.48) and Hispanics (OR=1.75, 95%CI=1.71-1.79) mostly suffered assaults, while the Whites suffered unintentional injuries (OR=2.78 95%CI=2.74-2.84); p<0.001. Optic nerve/ visual pathway injuries had the greatest association with very severe injury severity scores (OR=3.27, 95%CI=3.05-3.49) and severe Glasgow Coma Scores (OR=3.30, 95%CI=3.08-3.54); p<0.001. The mortality rate was 3.9%.

**Conclusions:**

Male preponderance and falls, motor vehicle accident-occupant, and struck by/against mechanisms agree with the previous reports. The identified demographic patterns underscore the need to develop group-specific preventive measures.

## Abstract (pt)

**Objetivo:**

O trauma ocular é uma causa importante e amplamente evitável de cegueira em todo o mundo. Nosso objetivo é estudar suas características nos EUA.

**Métodos:**

Revisão retrospectiva do *National Trauma Data Bank* (20082014). Todos os pacientes com trauma ocular foram identificados com códigos CID-9CM. Os dados coletados foram analisados estatísticamente e o teste t de student foi utilizado. As análises qui-quadrado e de regressão logística foram realizadas com o software SPSS. A significância foi estabelecida em p<0,05.

**Resultados:**

316.485 (5,93%) de 5.336.575 pacientes internados com trauma, apresentaram lesões oculares. A média (DP) de idade foi de 41,8 (23) anos. A maioria era do sexo masculino (69,4%). A distribuição raça/etnia foi branca: 66,1%, negra; 15,1% e hispânica: 12,3%. As lesões comuns foram orbitárias: 39,5% e contusões dos olhos/anexos: 34%. A maioria (58,2%) teve lesão cerebral traumática. Os mecanismos frequentes foram: quedas: 25,5%, acidente com veículos motorizados: 21,8% e acidentes atingidos por algo/contra algo: 17,6%. Os pacientes <21 anos apresentaram chance aumentada de lesões com corte/perfuração (RC=3,29; IC95% = 3,07-3,51) do que outras faixas etárias, aqueles entre 21-64 anos responderam por acidente automobilístico-ciclista: (RC=4,95; IC95% = 4,71-5,19) e aqueles > 65 anos foram vítimas de quedas (RC=16,75; IC 95% = 16,39-17,12); p<0,001. Os negros apresentaram maior probabilidade de lesões por arma de fogo (RC=3,24; IC95% = 3,10-3,39) do que outras raças/etnias e os hispânicos tiveram mais lesões de corte/perfuração (RC=2,01; IC95%= 1,85-2,18) enquanto os brancos tiveram mais quedas: (RC=2,3; IC95%= 2,3-2,4); p<0,001. Os negros (RC=3,41; IC95% = 3,34-3,48) e os hispânicos (RC=1,75; IC95% = 1,71-1,79) sofreram principalmente agressões e os brancos tiveram lesões não intencionais (RC=2,78; IC95% 2,74-2,84); p<0,001. Lesões do nervo óptico/via visual apresentaram maior associação com escore de gravidade de lesão muito grave (RC=3,27; IC95%= 3,05-3,49) e escores graves de Coma de Glasgow (RC=3,30; IC95%= 3,08-3,54); p<0,001. A taxa de mortalidade foi de 3,9%.

**Conclusões:**

Houve preponderância masculina a quedas, a acidentes com veículo motorizado atingidos por algo/contra algo conforme os relatos anteriores. Os padrões demográficos identificados realçam a necessidade de desenvolver medidas de prevenção específicas para os diferentes grupos.

## INTRODUCTION

Globally, ocular trauma is a major cause of unilateral blindness and visual impairment^(1-3)^. Prevent Blindness America estimated the total economic burden of visual impairment to be $139 billion (2011)^(4)^, and approximately 2.4 million annual US emergency department visits are related to ocular injuries^(5)^. Analyzing the contributing factors of ocular trauma are likely to be beneficial in developing preventive measures^(3,6)^.

Ocular trauma data are region specific, and hence, interventions need to be appropriately tailored. Differences may result from regional occupations and activities/exposures such as agriculture^(7,8)^, construction^(8)^, or exposure to firecrackers in China^(9)^, outdoor activities in the Mediterranean^(10)^, and assaults in urban areas^(11,12)^. Previous epidemiological studies have identified the patient characteristics and mechanisms of injury associated with blindness and visual impairment^(3,6,13)^. While studies in the US have focused on inpatient populations^(14)^, others have concentrated on ocular injuries resulting in permanent visual impairment^(3,6,15)^ or have studied small populations^(1,16)^. Studies utilizing large databases have included patients presenting with ocular injuries to emergency department (ED)^(5)^ and outpatient settings^(13)^; however, few have used a large database to evaluate ocular trauma in the setting of the major trauma admissions^(17)^. To this end, this study made use of the National Trauma Data Bank (NTDB), which contains the largest collection of trauma data in the United States and is under the auspices of the American College of Surgeons^(18)^. Ocular trauma is a significant cause of morbidity, and can result in lifelong disabilities that profoundly impact psychosocial development, employment potential, and independence^(19,20)^. Analysis of a large sample could help identify at-risk demographic groups and specific settings for injuries. Such information would be helpful in formulating policies aimed at preventing future ocular trauma.

## METHODS

This retrospective database-sourced study used de-identified data from the NTDB (2008-2014) following approval by the Institutional Review Board of Albert Einstein College of Medicine. Patients with ocular trauma were identified with reference to the Internal Classification of Diseases, Ninth Revision, Clinical Modification (ICD-9-CM). The included codes were as follows: open wound of ocular adnexa (870.0, 870.1, 870.2, 870.3, 870.4, 870.8, 870.9), open wound of eyeball (871.0, 871.1, 871.2, 871.3, 871.4, 871.5, 871.6, 871.7, 871.9), superficial injury (918.0, 918.2, 918.9), contusion of eye and adnexa (921.0, 921.1, 921.2, 921.3, 921.9, 364.41, 364.0, 364.3), foreign body on external eye (930.0, 930.1, 930.2, 930.8, 930.9), intraocular foreign body (360.59), burn confined to eye and adnexa (940.0, 940.1, 940.2, 940.3, 940.4, 940.5, 940.9), orbital injury (802.6, 802.7, 802.8, 802.9, 376.32, 376.33), injury to optic nerve and pathways (950.0, 950.1, 950.2, 950.3, 950.9), injury to other associated cranial nerves-III, IV, V, VI, VII, and shaken baby - (951.0, 951.1, 951.2, 951.3, 951.4, 951.9, 995.55), retinal injuries (362.81, 921.3, 361.00-361.33, 361.10, 363.63), vitreous hemorrhage (379.23), avulsion of globe (871.3), and hyphema (364.41). The mechanisms and circumstances of trauma were categorized using the following E codes: railway accidents (E800-E807), motor vehicle (E810-E29), water transport accidents (E830) air/space transport accidents (E840-E845), vehicle accidents not elsewhere classifiable (E846-E848), accidental poisoning (E850-E858, E860-E869), during surgical and medical care (E860-E876, E878-E879), accidental falls (E880-E888), other accidents (E890-929), drugs (E930-E949), self-inflicted or suicide (E950-E959), inflicted by other persons or assault (E960-E969), legal intervention (E970-E978), terrorism (E979), undetermined (E980-E989), and operations of war (E990-E999). Supplemental E codes were used to determine the external circumstances. Injury Severity Scores (ISS) and Glasgow Coma Scores (GCS) recorded by the ED and locations of all injuries were also documented. The ISS is a numeric stratification system that categorizes the degree of injury severity (range:1-75) and represents a spectrum from minor injury to increased risk of death. The mortality rate was determined by detailing dispositions including, home, facility transfer, nursing home, rehabilitation, hospice, death on discharge, left against medical advice, and unknown.

### Statistical analysis

The data collected included demographic information, type and mechanism of injury, intent and location, ISS, and GCS. Mean, median, and interquartile range were calculated for the continuous variables. The variables were categorized for logistic regression. The age groups were categorized into <21 years, 21-64 years, and >65 years, and the ocular injuries were categorized according to the ICD9-CM groups. Both ISS and GCS were classified according to the NTDB conventions: ISS: 1-8 (minor); 9-15 (moderate); 16-24 (severe); and >24 (very severe); GCS: 13-15 (mild traumatic brain injury [TBI]), 9-12 (moderate TBI), ≤8 (severe TBI). The association between the variables was determined using student’s t-test and Chi-squared test or Fisher’s exact test, as appropriate. Logistic regression analysis and determination of odds ratios and confidence intervals were performed to gauge the relative strength of the associations between the variables. The data were analyzed using SPSS software (Statistical Package for Social Science, IBM Corp, Armonk, NY), and graphs and tables were generated using Microsoft Excel (Microsoft Corp., Redmond, WA). All the *p*-values were two-tailed, and statistical significance was set at *p*<0.05.

## RESULTS

**Figure 1:**
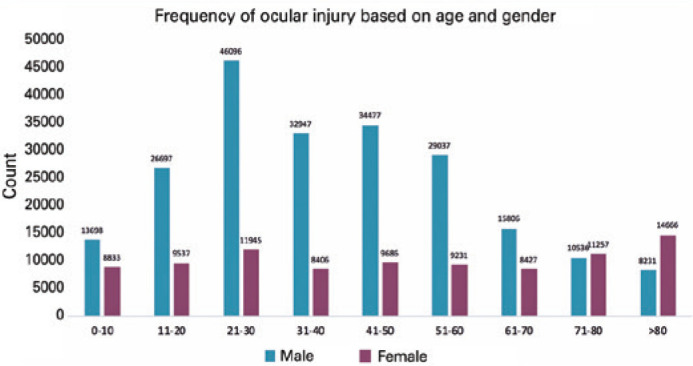
Frequencies of Ocular Trauma by Age and Gender, NTDB (2008-2014).

It was noted that 316,485 (5.93%) of the 5,336,575 admitted patients had ocular trauma. Their mean (SD) age was 41.8 (23) years, and a large proportion (61.7%) belonged to the economically active population of 2164 years. Men were injured more frequently (69.4%) than women (30.6%); besides, the men were younger, with a mean (SD) of 39.2 (20.5) years, when compared with the women, with a mean (SD) of 47.9 (27) years. In all the age groups, except for those >80 years of age, men outnumbered the women (Figure 1). The Whites were more frequently injured (66.1%) than the Blacks (15.1%) and “other” races (18.9%). The Hispanics constituted 12.3%. Level 1 trauma centers (38.5%) and the South (36.7%) reported the most of cases and the mean annual frequency was 45,212 (range: 37,218-52,891) (Table 1).

**Table 1:** Descriptive characteristics and demographic data of all patients with ocular trauma, National Trauma Data Bank (2008-2014)

Characteristics	Number	Percentage (%)	Characteristic	Number	Percentage (%)	Mean (SD)	Median (IQR)
Gender Male Female	219624 96861	69.4 30.6	Age (Years) 0-20 21-64 >65	58765 195156 55592	18.6 61.7 17.6	41.8 (23)	40.0 (23-58)
Race White Black Other	47660 209115 59710	15.1 66.1 18.9					
			Injury severity score 1-8 (Mild) 9-15 (Moderate) 16-24 (Severe) >24 (Very severe)	118501 86960 56576 40644	37.4 27.5 17.9 12.8	12.9 (10.4)	10 (5-17)
Ethnicity Hispanic	38884	12.3					
Year of injury 2008 2009 2010 2011 2012 2013 2014	37218 41583 43044 44082 49041 48626 52891	11.8 13.1 13.6 13.9 15.5 15.4 16.7					
			Glasgow coma score ≤8 9-12 13-15 Unknown	39517 12507 235535 28926	12.5 4 74.4 9.1	12.9 (3.7)	15.0 (13-15)
			Hospital stay (days) 1 day 2-3 days 4-6 days >6 days	79107 95102 62900 78809	25 30 19.9 24.9	6.2 (10.0)	3 (1-6)
Common injuries types Contusion eye/adnexa Orbital fractures Open wound adnexa Superficial Open wound eyeball Optic nerve/Visual pathway Related cranial nerves (III, IV, V, VI, VII and shaken baby)	107582 124887 73963 35056 29905 4045 7023	34 39.5 23.4 11.1 9.4 1.3 2.2					
			Locations of injury Street Home Public building Industry Recreation Residential institution Farm Mine Other Unspecified/Unknown	129154 85442 17323 6444 12155 10947 1753 162 15897 37208	40.8 27 5.5 2 3.8 3.5 0.6 0.1 5 11.8		
Traumatic brain injury	184124	58.2					
Intention of injury Unintentional Assault Self-inflicted Undetermined Unknown	231638 62595 3955 1496 16675	73.2 19.8 1.2 0.5 5.3					
			Region in USA Midwest Northeast South West Not applicable Unknown	65591 58868 116131 69270 1257 5368	20.7 18.6 36.7 21.9 0.4 1.7		
Mechanisms Falls MVT-occupant Struck by/against MVT motorcyclist MVT pedestrian Other Transport Firearms Cut/peirce Pedal cyclist Hot object Nature/environment Other Unknown	80678 68966 55580 16148 11500 8715 3587 14431 3237 1396 1472 50,775 16675	25.5 21.8 17.6 5.1 3.6 2.8 1.1 4.6 1 0.4 0.5 16.0 5.3					
			Level of trauma center Level 1 Level 2 Level 3 Level 4 Not Applicable	121714 58311 6557 654 129249	38.5 18.4 2.1 0.2 40.8		
Type of injury Penetrating Blunt Other Unknown	12361 261186 26263 16675	3.9 82.5 8.3 5.3	Eye Protection - Work	148 (of 8359)	1.8		
			Eye Protection - Recreation Substance abuse (Alcohol/Drugs)	46 (of 12155) 31,604	0.4 9.98		
			Mortality	12233	3.9		

N/A= not applicable; SD= standard deviation; IQR= interquartile range.

Most ocular injuries occurred in the 21-64-year-old age group and men outnumbered women in all groups, except for those aged >80 years.

**Figure 2:**
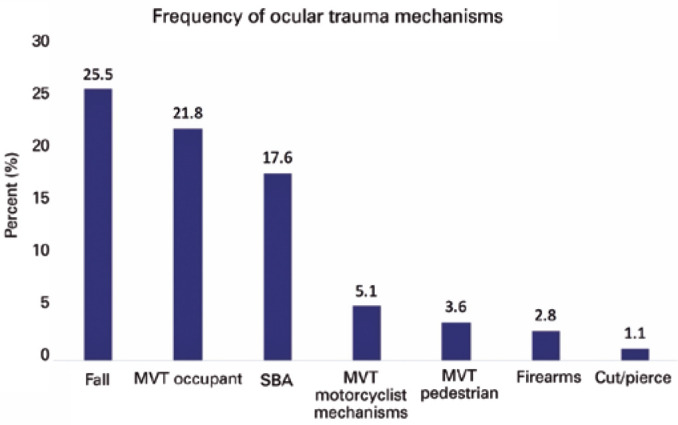
Mechanisms of Ocular Trauma, NTDB (2008-2014).

**Figure 3:**
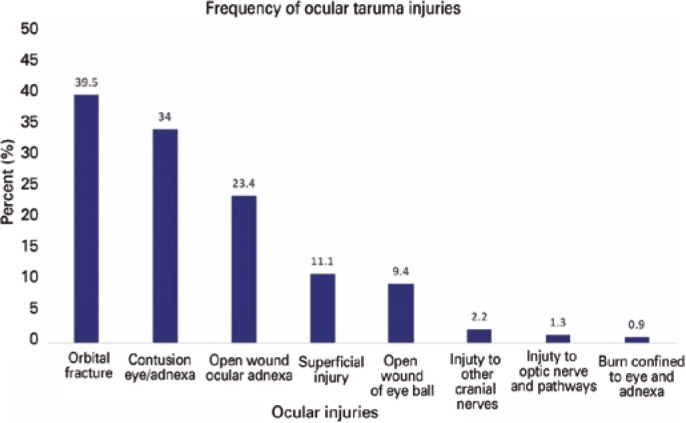
Types of Ocular Injuries, NTDB (2008-2014).

Most ocular trauma was blunt (82.5%). The intentions, in descending order, were unintentional (73.2%), assault (19.8%), and self-inflicted (1.2%). The frequent mechanisms were falls (25.5%), motor vehicle traffic-occupant (MVT-occupant) (21.8%), and struck by/against (SBA) (17.6%) (Figure 2). The most common injuries were orbital (39.5%), eye/adnexal contusions (34%), and open ocular adnexal wounds (23.4%) (Figure 3). Open eyeball wounds (9.4%) and optic nerve/pathway injuries (1.3%) were infrequent. However, associated TBI was documented in 58.2%. The mean (SD) ISS was 12.9 (10.4), and most of the patients (67.6%) sustained minor-to-moderate injuries. The mean (SD) GCS was 13.1 (3.8), and mild TBI was the largest sub-group (47.1%).

Frequent mechanisms were falls, MVT-occupant and stuck by or against injuries. MVT= motor vehicle traffic; SBA= struck by or against.

Common of ocular injuries were orbital injuries, contusions of the eye or adnexa, and open wound ocular adnexa.

The most common locations were the street (40.8%) and home (27%). When broken into the four census regions, 36.7% injuries were reported from the South, 21.9% from the West, 20.7% from the Midwest, and 18.6% from the Northeast. In work-related injuries such as those sustained in industries, mine, or farm locations, eye protection was used in 1.8% of the cases, and in recreational activities, it was used in only 0.4% of the cases (total, 2.2%). Substance abuse was associated with 10% of the injuries. The mortality rate was 3.9% (Table 1).

### Comparative analysis

#### Differences in demographic and regional patterns

**Figure 4:**
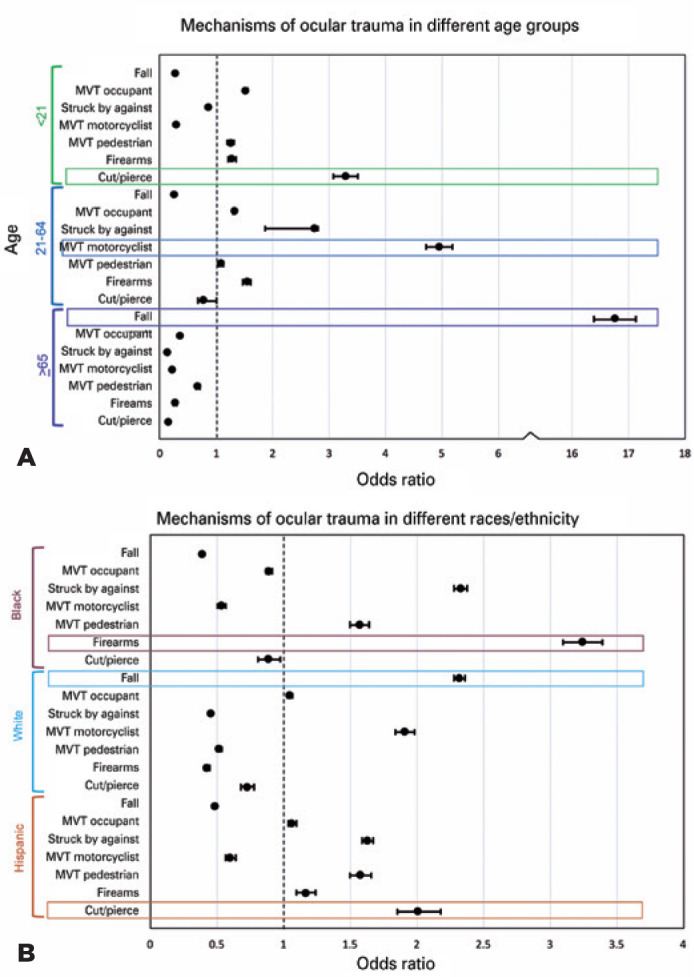
Summary of Regression Analysis of Mechanisms Association with (A) Age group and (B) Race/Ethnicity, NTDB (2008-2014).

Logistic regression analysis of the three broad age groups revealed that the youngest group (<21 years) had greater odds than the other age groups for cut/pierce mechanism of injury (OR=3.29, 95%CI=3.07-3.51), and recreational locations (OR=2.87, 95%CI=2.77-2.98); p<0.001. On the other hand, those in the age group of 21-64 years were more prone to MVT motorcyclist injuries (OR=4.95, 95%CI=4.71-5.19) and industrial locations (OR=6.27, 95%CI=5.76-6.83); p<0.001. The elderly (>65 years) had the greatest odds of falling (OR=16.75, 95%CI=16.4-17.12) and sustaining injury in residential institutions (OR=4.57, 95%CI=4.38-4.76) and home locations (OR=3.80, 95%CI=3.73-3.88); p<0.001) (Figure 4A). The intentions varied with age; those in the 21-64 age group had greater odds of assault (OR=3.50, 95%CI=3.43-3.58), while those in the <21 (OR=1.37, 95%CI=1.34-1.40) and >65 age groups (OR=8.84, 95%CI=8.48-9.22) had the greatest odds of unintentional injury; p<0.001.

(A) Falls were the most common mechanism and most likely to occur in the oldest age group, while MVT-cyclist and struck by or against most likely to occur in the 21-64-year-old age group and cut/pierce in the youngest group, when compared to the other groups. (B) Falls were the most likely to occur in white patients, firearms in black patients, and cut/ pierce injuries in Hispanic patients.

Men had greater odds of SBA injuries (OR=3.38, 95%CI=3.94-3.47) and industrial location (OR=7.87, 95%CI=7.06-8.77), while women had greater odds of sustaining falls (OR=2.94, 95%CI=2.89-2.99) and being injured at home (OR=2.07, 95%CI=2.03-2.10); p<0.001. Men were most likely to suffer assault injuries (OR=3.05, 95%CI=2.98-3.12), and women were most likely to suffer unintentional injuries (OR=3.02, 95%CI=2.95-3.08); p<0.001.

Analysis based on race/ethnicity revealed that the Blacks were more likely to sustain injury from firearms (OR=3.24, 95%CI=3.1-3.39), and in the streets (OR=1.11, 95%CI=1.08-1.13); p<0.001. In contrast, the Whites had greater odds of falls (OR=2.32, 95%CI=2.28-2.37; p<0.001). Although the Whites had the greatest odds of farm location (OR=3.67, 95%CI=3.19-4.24; p<0.001) when compared with other races/ethnicities, they also had higher odds of mines, home, recreation, and residential institution locations than the Blacks and Hispanics. The Hispanics had the greatest odds of cut/pierce injuries (OR=2.01, 95%CI=1.85-2.18) and industrial locations (OR=2.25, 95%CI=2.12-2.38); p<0.001, (Figure 4B). With respect to intentions, the Whites were most likely to be injured unintentionally (OR=2.79, 95%CI=2.74-2.84), while the Blacks (OR=3.41, 95%CI=3.34-3.48) and Hispanics (OR=1.75, 95%CI=1.71-1.8) were most likely to face assault injuries; p<0.001. The Whites suffered more from self-inflicted injuries than those from other races/ethnicities (OR=1.74, 95%CI=1.61-1.87; p<0.001).

Of the 31,604 cases of substance abuse (alcohol/ drugs)-associated injuries, most were men (78.7%), Whites (61.4%), in the 21-64 age group (83.9%), and unintentional trauma (63.9%). For the entire group, substance abuse was more associated with SBA (OR=1.64, 95%CI=1.55-1.75; p<0.001) than other injuries. However, the association with mechanisms differed between the demographic groups. For the groups most associated with substance abuse, men were more commonly injured by MVT-occupant (OR=1.39, 95%CI=1.33-1.45; p<0.001) than other mechanisms, the 21-64 age group by MVT-pedestrian (OR=1.40, 95%CI=1.291.53; p<0.001), and Whites by MVT-motorcycling trauma (OR=1.89, 95%CI=1.76-2.02; p<0.001).

The patients from the Northeast had greater odds of falls (OR=1.99, 95%CI=1.95-2.03), the Midwest, SBA (OR=1.07, 95%CI=1.05-1.10), the South, MVT-occupant (OR=1.46; 95%CI=1.43-1.48), and the West, MVT-pedestrian (OR=1.53, 95%CI=1.47-1.6); p<0.001. Additionally, firearm injuries were most likely to occur in the South (OR=1.3; 95%CI=1.24-1.36; p<0.001), and in this region, the Whites had a greater likelihood of firearm injuries than any other race/ethnic group (OR=1.09; 95%CI=1.0-1.19; p=0.05).

#### Severity of ocular trauma

**Figure 5:**
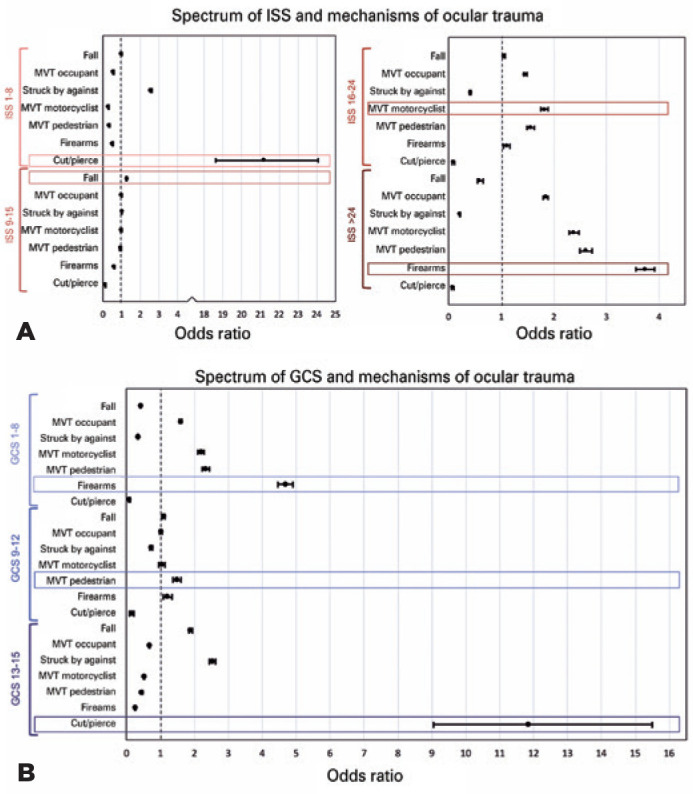
Summary of Regression Analysis of Mechanisms Association with (A) Injury Severity Score and (B) Glasgow Coma Score, NTDB (2008-2014).

The mechanisms with the highest odds of very severe injury severity (ISS) were firearms (OR=3.73, 95%CI=3.56-3.91), followed by MVT-pedestrian (OR=2.61, 95%CI=2.49-2.72) and MVT motorcyclist (OR=2.38, 95%CI=2.29-2.47); p<0.001. Those with the lowest ISS were cut/pierce (OR=21.83, 95%CI=18.6321.09) and SBA (OR=2.57, 95%CI=2.52-2.62); p<0.001. Although pedal cyclists had the greatest odds of sustaining TBI (OR=2.13, 95%CI=2.01-2.25; p<0.001), when broken down to the levels of TBI as reflected by the GCS, the mechanisms were different. Firearms (OR=4.77, 95%CI=4.47-4.89) and MVT-pedestrian (OR=2.33, 95%CI=2.23-2.43) had the greatest odds of severe TBI. Like ISS, cut/pierce (OR=11.84, 95%CI=9.04-15.49) and SBA (OR=2.53, 95%CI=2.45-2.61) had the greatest odds of mild TBI (Figure 5).

(A) Cut/pierce injuries were most associated with mild injury severity and firearms, the most severe injury severity. (B) Similarly, cut/pierce injuries were most associated with the mild levels of TBI and firearms, the most severe level of TBI. ISS= injury severity score; GCS= Glasgow coma score; TBI= traumatic brain injury.

Burns confined to the eye and adnexa were most associated with the lowest ISS (OR=6.14, 95%CI=5.616.72; p<0.001). Open wounds of the adnexa (OR=1.36, 95%CI=1.33-1.39) and open globe injuries (OR=1.29, 95%CI=1.25-1.34) were associated with the mildest TBI; p<0.001. However, optic nerve/visual pathway injuries had the greatest odds of very severe ISS (OR=3.27, 95%CI=3.05-3.49) and severe TBI (OR=3.30, 95%CI=3.08-3.54); *p*<0.001. Self-inflicted injuries had the greatest odds of TBI (OR=2.39, 95%CI=2.22-2.58; p<0.001). These patients had the greatest odds of dying from their injuries (OR=7.66, 95%CI=7.09-8.27; *p*<0.001).

## DISCUSSION

Approximately 6% of the admitted trauma patients had ocular trauma. Most patients were young, men, Whites, and from the South. The common mechanisms, in descending order, were falls, MVT-occupant, and SBA. The common locations were the street and home. The frequent injuries were orbital and eye/adnexal contusions. Although most were considered mild, the majority of injuries were associated with TBI. Orbital injuries were most associated with severe ISS, while optic nerve/ visual pathway injuries were most associated with very severe ISS and severe GCS.

The mechanisms, locations, intentions, and levels of ISS and GCS varied with age, gender, and race/ethnicity. Those in the youngest age group (<21 years) had greater odds of sustaining minor injuries, cut/pierce mechanisms, and at home location. In contrast, the working age group (21-64 years) had greater odds of very severe injuries, MVT motorcyclist mechanism, and the street location. Finally, the elderly (>65 years) sustained moderate and severe injuries, falls mechanism and home location. These differences were also noted between races/ethnicities. The Blacks suffered more from firearm injuries and street location, Whites from falls and farming location, and Hispanics from cut/pierce injuries and industry location when compared with other races/ethnicities. With respect to intention, the Blacks and Hispanics were most likely to be assaulted, and the Whites were most likely to suffer unintentional injury. Our findings regarding the mechanisms and intentions agree with the results from previous studies, which had also noted propensities to assault among the Hispanics and Blacks^(13,21-23)^.

Previous studies have evaluated the types of injuries and mechanisms of ocular trauma and have related them to demographic details^(3,5,6,17,23)^. Scruggs et al.^(17)^ used weighted NTDB (National Sample Program) data to evaluate 28,340 cases. Similar to our findings, they also identified that the most common injuries were orbital; a young age and male preponderance were also established. However, the mean age was younger (38.2 years), and the most frequent mechanisms were related to motor vehicles. Falls were most frequent in our study. Although these disparate findings might have resulted from the use of different databases, they may also indicate that fall-related injuries are increasing in the aging US population^(23)^. Haring et al.^(5)^, using a different weighted database (Nationwide ED Sample, NEDS), evaluated 4,317,164 cases and found a similar male preponderance but a younger age (33.8 years) than our study (41.8 years). Furthermore, their most common mechanisms were foreign bodies, followed by falls. Again, the difference from our findings can be attributed to the variations in sourcing the data and in the populations. Haring’s study of ED patients included conditions that are routinely dealt with on an outpatient basis, while our study focused on admitted major trauma patients. Using logistic regression, they found that men, older patients, SBA injuries, and multiple injuries were most associated with inpatient admission^(5)^.

The analyses performed in this study revealed that locations were also associated with demographic groups. May et al.^(3)^, in their study of serious eye injuries using the United States Eye Injury Registry, found that the most common location of injury was the home, followed by industry. Streets and recreation/sport tied for the third place. This study determined that the street was the most common location, followed by home and public buildings. May’s study identified that the street accounted for only 9%, while our study found motor vehicle/ cycle or pedestrian accidents accounted for about 40% of the injuries. Their finding of poor compliance with regulations regarding eye-protective glasses in the workplace and recreation (2%) agreed well with our finding of 2.2% compliance. This observation has implications for focusing on campaigns that promote eye safety compliance in the workplace and during sports activities.

Our study used regression models to elucidate the patterns of association between mechanisms, locations, intent, severity indices (ISS and GCS), and demographics. To our knowledge, these have previously not been reported in largescale ocular trauma studies (Figures 4 and 5). The mechanisms of ocular injury have been analyzed in earlier studies using frequencies^(5,17,23)^. The frequencies of intent of ocular injury have been reported previously^(5,6)^; however, our analyses unearthed demographic differences in the intentions of injury. Assaults were more likely in the working age group (21-64 years) and Blacks and Hispanics. Unintentional injuries were most likely in Whites and in the youngest (<21 years) and oldest (>65 years) age groups. Self-inflicted injuries were most likely in Whites and were most commonly associated with optic nerve/visual pathway injuries, very severe ISS, and severe TBI. Consequently, the Whites suffered the highest inpatient mortality.

This study has several strengths, not the least of which is its scope. We identified the at-risks groups and the demographically associated circumstances of their injuries, including mechanisms, substance abuse relations, locations, and intention, which could be used to create guidelines for preventive strategies. TBI is known to be associated with ocular trauma^(24-27)^, which was confirmed in this study. The concurrence of TBI may manifest in multiple ways and contribute to shortand long-term disability. These complications must be considered during post-discharge multidisciplinary care and rehabilitation. Additionally, we considered the impact of intention; few reports have evaluated the impact of intention on the severity of injury using ISS and TBI and the resulting mortality. In a report of intentions in pediatric ocular injuries using the same NTDB data, Gise et al.^(28)^ also noted a stronger association of self-inflicted injury with TBI and mortality than the other intentions. These outcomes likely reflect the associations that can be generated using similar analytical models for all trauma. Indeed, Deng et al., in their studies of firearms, related the TBI using weighted NTDB (NSP) data (2003-2012) and likewise found that self-inflicted injuries had the highest levels of TBI and ISS, and consequently, higher rates of mortality^(29,30)^.

This study also has some limitations, the most important one being its retrospective design and database sourcing. The results can only reflect the integrity of the submitted data. Furthermore, detailed ophthalmic findings and outcomes were not available, which makes comparison with population-based reports difficult. The data used were not the weighted NTDB (NSP) and may hence represent variable annual trauma center and regional contributions. Moreover, the full scope of ocular trauma, especially minor injuries that are encountered in outpatient settings, are not represented. This issue might lead to an underestimation of the ocular trauma in the US, while focusing on patients with major injuries alone. Lastly, NTDB (2008-2014) used ICD-9CM codes, which are less precise than the current ICD-10CM codes.

This study confirmed that young and male patients were disproportionally affected by ocular trauma and most patients were in the economically active age group. We identified demographic variations in mechanisms, location, association with substance abuse, intention of injury, and association between intention and types of ocular injuries and indices of severity. An analysis of more current datasets that confirm our results could provide a foundation for the design and implementation of focused interventional measures.
